# Editorial: Psychosocial and bioethical challenges and developments for the future of vascularized composite allotransplantations

**DOI:** 10.3389/fpsyg.2023.1186113

**Published:** 2023-04-20

**Authors:** Martin Kumnig, Nikolas R. Hummel, Sheila G. Jowsey-Gregoire, Kevin J. Zuo, Elisa J. Gordon, Simon G. Talbot

**Affiliations:** ^1^Department of Psychiatry, Psychotherapy, Psychosomatics and Medical Psychology, Center for Advanced Psychology Transplantation Medicine (CAPTM), Medical University of Innsbruck, Innsbruck, Austria; ^2^Department of Psychiatry and Psychology, Mayo Graduate School of Medicine, Mayo Clinic Rochester, Rochester, MN, United States; ^3^Division of Hand and Upper Extremity Surgery, Department of Orthopaedic Surgery, Beth Israel Deaconess Medical Center, Boston, MA, United States; ^4^Department of Surgery, Vanderbilt University Medical Center, Nashville, TN, United States; ^5^Center for Biomedical Ethics and Society, Vanderbilt University Medical Center, Nashville, TN, United States; ^6^Division of Plastic Surgery, Brigham and Women's Hospital, Boston, MA, United States; ^7^Harvard Medical School, Boston, MA, United States

**Keywords:** vascularized composite allotransplantation, patient evaluation, follow-up, multicenter research approach, informed consent, patient-provider communication/information

Vascularized composite allotransplantation (VCA) remains a relatively new field of medicine and transplantation, with a history of successful transplantation starting fewer than 25 years ago, and including more than 150 transplants to date. The potential to restore lost function and to improve quality of life for individuals with severe impairments propelled early research focused on the technical and immunological aspects of these procedures. As more cases are studied and advances in the field are made, the importance of psychosocial and bioethical considerations has become increasingly relevant (Kinsley et al., 2020, 2022).

The aim of this Research Topic is to create a foundation of high quality articles that address psychosocial and bioethical challenges and developments in the evolving field of VCA with viewpoints driven by research and clinical experience, across the varied types of VCA (e.g., hand, face, and uterus transplantation). Furthermore, this review summarizes recent discussions and conclusions reached by the Chauvet Workgroup, an international multicenter and multidisciplinary platform to bring together expertise and to learn from VCA cases performed worldwide. The Chauvet Workgroup's mission is to improve understanding of psychosocial and bioethical factors in VCA through an open global platform for all interested parties with no defined ownership and broad collaboration in research and clinical practice. The Chauvet Workgroup holds great value and potential because of its core principle of collaborative international research and clinical approach to the psychosocial evaluation in VCA. The Chauvet Workgroup has been endorsed by both the International Society for VAC (ISVCA) and American Society for Reconstructive Transplantation (ASRT) with regular presentations at their meetings to summarize activities of the biennial workshops (Jowsey-Gregoire et al., 2016; Kumnig and Järvholm, 2022).

Psychosocial aspects of VCA span the lifecycle of VCA. Initial evaluation and assessment of candidates participants, optimization of psychosocial variables, peri-operative support, and preparation and management of post-transplantation changes and adjustment are just some of the areas of research and interest (Kinsley et al., 2021). Success in VCA requires a match between the operation and patient. We often consider this “appropriate patient selection” (Kumnig et al., 2012, 2014). However, psychosocial research also allows, perhaps more importantly, optimization of potential candidates to ensure adequate support and interventions to expand candidacy. Thus, psychosocial evaluation should include both a comprehensive evaluation combined with follow-up protocols and supportive interventions before (considering risk-benefit issues) and after VCA (for example, dealing with the graft in daily life, etc.) (Jowsey-Gregoire and Kumnig, 2016; Kumnig and Jowsey-Gregoire, 2016).

In addition to the psychosocial aspects that span the lifecycle of VCA discussed herein, bioethical concerns need to be considered as fundamental elements of patient evaluation and follow-up protocols that provide the foundation for successful VCA (Gordon and Siemionow, 2009; Gordon et al., 2011). For example, a variation in clinical practices pertaining to candidate evaluation and informed consent processes mean that some candidates my receive less rigorous evaluation or information from others, which may undermine their consideration for candidacy or shared decision making. Thus, VCA organizations providing bioethical and policy oversight of the organ transplant system should establish standards to ensure that all VCA candidates/recipients are treated equitably (Gacki-Smith et al.).

While VCA raises bioethical concerns pertaining to all principles, VCA challenges some principles more than others. Specifically, respecting patients' self-determination (autonomy) is particularly challenging given the limitations of data on long-term psychosocial and clinical outcomes. Given the relatively small number of patients receiving VCA organs, the VCA field faces challenges in amassing sufficient information about the risks and benefits that VCA recipients commonly experience. Accordingly, VCA candidates may find it difficult to make meaningful informed treatment decisions and provide informed consent to pursue VCA.

This Frontiers Research Topic demonstrates the need for more collaborative, multicenter research in order to optimize these highly complex medical procedures, and to bring together expertise and to learn from as many of the VCA cases performed as possible. This Research Topic builds on the work of many research and clinical teams and aims to provide a unifying framework to evolve research on psychosocial issues. The authors describe the relevant psychosocial and bioethical considerations for the formation of an international research platform and outline its vision and current process, based on international research collaborations including most predominantly that of the Chauvet Workgroup (Kumnig et al., 2022). Additionally, recent efforts of the Organ Procurement and Transplantation Network (OPTN) and the United Network for Organ Sharing (UNOS) support these developments. Finally, this paper collection identifies key issues that should be addressed in future research.

*Statistics on this Research Topic*: This Research Topic was open between May 2022 and February 2023. After a rigorous and constructive reviewing process of numerous submissions, ultimately 10 articles by 53 authors were accepted. Since its inception, this Research Topic has received over 7,200 views (as of March 2023).

*Overview of this Research Topic*: To cover these themes, this Frontiers Research Topic consists of the following types of manuscripts: original research, review and mini review, conceptual analysis, brief research report, and opinion. The submission of original articles was particularly appreciated, presenting recent research innovations in this field, including results of qualitative research initiatives to investigate relevant psychosocial outcome predictors.

## Author contributions

All authors listed have made a substantial, direct, and intellectual contribution to the work and approved it for publication.
